# High Progesterone levels in the beginning of ICSI antagonist cycles and clinical pregnancy: still a concern?

**DOI:** 10.5935/1518-0557.20170004

**Published:** 2017

**Authors:** Tatiana R Panaino, Joyce B da Silva, Maria Augusta T de Lima, Paloma Lira, Patricia C Arêas, Ana Cristina A Mancebo, Marcelo M de Souza, Roberto A Antunes, Maria do Carmo B de Souza

**Affiliations:** 1 Clinica Fertipraxis, Rio de Janeiro, RJ, Brazil

**Keywords:** progesterone, antagonists, hyperstimulation, *in vitro* fertilization

## Abstract

**Objective:**

In controlled ovarian hyperstimulation (COH) using antagonist cycles, an incomplete luteolysis could happen after an inefficient previous luteolysis. Since antagonist cycles are frequent today, this study aims to access the impact of serum progesterone in the beginning and at the end of stimulation, and pregnancy outcomes.

**Methods:**

single-center cohort study, 461 fresh embryo transfers in ICSI antagonist cycles. Serum progesterone levels was measured in the beginning of COH (P4i) and on hCG day (P4f) using threshold values of 1.5ng/mL. Four groups were created: Group 1, P4i and P4f ≤ 1.5; Group 2, P4i ≤ 1.5 and P4f > 1.5; Group 3, P4i > 1.5 and P4f ≤ 1.5 and Group 4, P4i and P4f > 1.5. The clinical pregnancy rate (CPR) and live birth rates (LBR) were the primary outcomes.

**Results:**

The number of cycles per group was: 393, 51, 6 and 11, respectively. Group 1 was considered the expected normal, while group 4 represented the persistence of higher levels. There was no difference in age, basal FSH and Estradiol, days of stimulation endometrium thickness and total amount of gonadotropins between group 1 *versus* group 4. However, significant differences occurred in embryological and clinical outcomes between these 2 groups.

**Conclusion:**

The impact of serum progesterone in the beginning of stimulation and pregnancy outcomes is a matter of concern. Basal elevated levels could help identify patients that will repeat it on hCG day, being probably a marker to define a freeze-all strategy to these cycles.

## INTRODUCTION

Median menstrual cycle length of 28 days was indicated by Treloar *et al*. (1967) with a normal range between 25 and 35 days. This interval may show a wider variability between and within individuals, both immediately following menarche and preceding menopause. Usually, the length fluctuations of the follicular phase are primarily responsible for the variations in cycle length noted among women. Luteal phase is more constant, lasting between 10 and 16 days in 95% of cycles.

The spontaneous ovulatory menstrual cycle is regulated by complex interactions of the hypothalamic-pituitary axis, the ovaries and the genital tract. During the follicular phase, estrogen levels rise in parallel to growth of a dominant follicle. Its production occurs in the follicle's granulosa cells, site of FSH receptor expression. FSH induces aromatase and consequent expansion of the antrum in growing follicles. The dominant follicle will then initiate expression of LH receptors, that lead to the beginning of secretion of small quantities of progesterone. The preovulatory progesterone secretion, although limited, probably exerts positive feedback on the estrogen-primed pituitary to either cause or augment LH release. LH peaks 10 to 12 hours before ovulation and current studies suggest that it happens in response to this increased progesterone and prostaglandin production by the cumulus cells (Cunningham *et al*., 2010).

Following ovulation, the corpus luteum develops from the remains of the dominant follicle with an increased capacity to produce progesterone, which peaks during the midluteal phase. In the absence of pregnancy, the corpus luteum will regress 9 to 11 days after ovulation by mechanisms that remain unclear. This so called luteolysis, consists of a dramatic drop in circulating estradiol and progesterone levels due to apoptotic cell death (Vaskivuo *et al.*, 2002). These events enable follicular development and ovulation during the next ovarian cycle. When conception occurs, the human corpus luteum is rescued from luteolysis by elevating levels of trophoblast-derived hCG.

In controlled ovarian hyperstimulation (COH) using antagonist cycles, an incomplete luteolysis could happen after an inefficient previous luteolysis because the GnRH antagonists added later in the cycle (at stimulation day 5-6) for premature LH surge inhibition. Kolibianakis *et al*. (2004) suggests that elevated serum progesterone (> 1,6 ng/mL) levels on day 2 could decrease the likelihood of pregnancy. These authors stated that these cycles should be postponed for 1-2 days. Only then should we consider beginning the stimulation protocol. This way, we could avoid those elevated progesterone levels. The last 12 years have brought more data, and the discussion turned to the impact of higher progesterone levels on the hCG day. Compelling evidence support that it is associated with a decreased probability of pregnancy when a fresh embryo transfer is performed (Labarta *et al*., 2011). In contrast, in frozen/thawed and oocyte donation cycles, in which embryos originated from women with progesterone elevation during COH, no decrease in pregnancy rates were detected when embryos were transferred to an endometrium not exposed to elevated progesterone levels (Bosch, 2010; Venetis *et al*., 2013; 2015). As antagonist cycles are frequent today, this study turns to access the impact of serum progesterone in the beginning and at the end of stimulation and clinical pregnancy rates outcomes.

## MATERIAL AND METHODS

This is a single-center retrospective cohort study of 461 fresh embryo transfers in ICSI antagonist cycles. We enrolled in the study 418 patients ≤ 39 years, who were undergoing IVF/ICSI cycles from January 2004 to Jan 2015.

Ovarian stimulation was carried out using only recombinant follicle-stimulating hormone (rFSH; Gonal-F^®^: Merck Serono, Geneva, Switzerland) in 184 cases. In 244 cases, we used highly purified human menopausal gonadotropin (HMG; Menopur^®^: Ferring Pharmaceuticals, Switzerland), added to rFSH; and in 33 cases only HMG was used. Gonadotropins were initiated on day 2 of the cycle. Doses varied from 150 to 300 IU/day, depending on the patient's age, body mass index, ovarian pattern, menstrual cycles, basal hormones, and response to previous COH. Follicle growth was assessed by vaginal ultrasound. A daily dose of 0.25 mg of GnRH antagonist (Cetrorelix^®^; Cetrotide: MerckSerono, Geneva, Switzerland) administration was initiated when the leading follicle reached 14 mm and continued until the day of human chorionic gonadotropin (hCG) administration. Oocyte maturation was trigged by subcutaneous administration of hCG (rhCG; Ovidrel^®^: Merck Serono, Geneva, Switzerland) when at least one follicle had reached 18 mm in diameter and two had reached 16 mm. Transvaginal oocyte retrieval was scheduled 34 to 36 hours later. Luteal phase supplementation was started on oocyte retrieval day and consisted of 600 mg per day of natural micronized progesterone administered intravaginally, in three divided doses (Micronized progesterone; Utrogestan^®^:Farmoquimica, Brazil), and was maintained until the ß-HCG serum assay and, if positive, it was continued until 12 weeks of gestation.

All 461 cycles were divided according to their serum progesterone levels in the beginning of COH (P4i) and on hCG day (P4f) using threshold values of 1.5ng/mL. Four groups were created: Group 1, P4i and P4f ≤ 1.5; Group 2, P4i ≤ 1.5 and P4f > 1.5; Group 3, P4i > 1.5 and P4f ≤ 1.5 and Group 4, P4i and P4f > 1.5

Clinical pregnancy rates (CPR) and live birth rates (LBR) were the primary outcomes. CPR was defined by ultrasound visualization of a gestational sac at 6 weeks of pregnancy with positive embryo heart rate on ultrasound per embryo transfer. LBR was the number of deliveries that resulted in at least one live born neonate per embryo transfer (Zegers-Hochschild *et al*., 2010).

The method used for oocyte and sperm preparation, and embryo development assessment has been described previously (Souza *et al*., 2009).

The following patient characteristics were evaluated: age, basal progesterone (P4), estradiol and FSH levels, and infertility etiology. Other parameters included: the dose and duration of gonadotropins administration; endometrial thickness, number of retrieved oocytes, number of metaphase II oocytes, fertilization and cleavage rates, number of available and transferred embryos, implantation rate, biochemical/clinical pregnancy rates and LBR.

All patients signed an informed consent giving permission to use their information on scientific papers, before starting their own cycles. Because the data were analyzed retrospectively and anonymously, no other permission was asked to an Ethics Committee.

Clinicians selected an appropriate protocol for each patient on a case-by-case basis per patient characteristics. There was no delay in beginning the cycles based on initial P4 levels. Serum progesterone levels were measured using mostly electrochemiluminescence immunoassay. There is consistency on our progesterone assays because 80% of them came from the same Clinical Laboratory and the techniques were sequentially validated whenever changed (Patton *et al*., 2014). To test our hypothesis that elevated serum progesterone levels during COH decreased pregnancy rates, we elected a cut-off value of > 1,5 ng/mL. Either in the beginning or on the hCG day. This threshold was based on previous studies (Pritsivelis *et al*., 2004; Venetis *et al*., 2013).

For the statistical analysis, Student's t-test was used for comparison of numerical variables and the chi-square test for qualitative data. Anova was used to compare mean values, and post hoc tests were done using Dunnett's multiple comparison tests. The difference was considered statistically significant when *p* < 0.05.

## RESULTS

The analysis of 461 fresh cycles from 418 patients that fulfilled the inclusion criteria of the progesterone assay both at the beginning of the cycle and on hCG day were divided into four groups according to the P4 level (ng/mL). Resulting groups were as follows: group 1: 393 cycles; group 2: 51 cycles; group 3:11 cycles and group 4: 6 cycles.

All four groups presented the following infertility etiology in groups 1,2,3 and 4 respectively: male factor infertility: 32,8%, 47,0%, 36,3% and 66,6%. Female factor infertility: 28,2%, 21,5%, 36,3% and 33,3%. Unexplained infertility: 33,8%, 25,4%, 18,1% and 0%. Mixed factors: 3,8%, 3,9%, 9,0% and 0%. Other causes: 1,2%, 1,9%, 0% and 0%.

To look for potential differences, Group 1 was considered the expected normal, while group 4 was the target of the study. Table 1 represents the main demographics and stimulation parameters, such as: female age, days of stimulation, total amount of gonadotropins and basal hormonal profile in each group.

**Table 1:** Main demographics and stimulation parameters of each group.

Parameters	Group 1	Group 2	Group 3	Group 4	p-value
	P4i ≤ 1.5 and P4 f ≤ 1.5	P4i ≤ 1.5 and P4 f > 1.5	P4i > 1.5 and P4f ≤ 1.5	P4i > 1.5 and P4 f > 1.5	
Nº patients	350	51	11	6	
Nº Cycles	393	51	11	6	
Female age	34.14 ± 2.92 (25 - 38)	33.58 ± 2.87 (28 - 38)	33.81 ± 3.06 (27 - 38)	32.83 ± 2.40 (29 - 35)	NS
Basal FSH (UI/ml)	6.91 ± 5.74	5.96 ± 2.95	6.58 ± 2.47	5.00 ± 1.92	NS
Basal Estradiol (pg/mL)	72.95 ± 79.13	72.12 ± 56.93	54.36 ± 25.10	64.16 ± 55.32	NS
Basal P4 (ng/mL)	474.48 ± 260.81^abc^	751.41 ± 317.72_ad_	2844.18 ± 1477.10^cd^	3586.16 ± 2223.30^b^	^abcd^< 0.05
N. follicles ≥ 18 hCG day	2.76 ± 1.60^ab^	4.39 ± 3.16^a^	2.45 ± 1.29	5.00 ± 1.54^b^	^ab^< 0.05
Total gonadotropins (IU)	2016.40 ± 717.25^a^	2228.92 ± 701.74	2844.18 ± 1477.10^a^	2416.66 ± 358.35	^a^< 0.01
Duration of stimulation	9.65 ± 1.59	10.23 ± 1.54	9.72 ± 2.05	10.00 ± 0.63	NS
Endometrium thickness ET day	10.58 ± 5.06	10.43 ± 2.14	10.40 ± 2.88	10.51 ± 1.32	NS

NS: Not significant.

Table 2 represents the embryological and clinical outcomes.

**Table 2:** Embryological and clinical outcome parameters for each group.

Parameters	Group 1	Group 2	Group 3	Group 4	p-value
	P4i ≤ 1.5 and P4 f ≤ 1.5	P4i ≤ 1.5 and P4 f > 1.5	P4i > 1.5 and P4f ≤ 1.5	P4i > 1.5 and P4 f > 1.5	
Nº Patients	350	51	11	6	
Nº Cycles	393	51	11	6	
Oocytes retrieved	7.62 ± 4.71^ab^	11.47 ± 5.91^a^	7.54 ± 5.42^c^	18.16 ± 10.36^bc^	^abc^< 0.05
MII oocytes retrieved	4.85 ± 3.45^a^	7.52 ± 4.73^ab^	3.90 ± 3.80^b^	10.33 ± 7.33	^ab^< 0.05
Maturation rate (M2/oocytes retrieved)	63% (1905/2997)^a^	65% (384/585)^b^	52% (43/83)^ab^	57% (62/103)^ab^	^ab^< 0.03
Fertilization rate	80%	77%	72%	71%	NS
Embryos cleavage rate	99%	99%	99%	98%	NS
Nº embryos transferred	2.14 ± 0.73	2.60 ± 0.77	2.09 ± 0.83	2.50 ± 0.83	NS
Embryos ≥ 8CG1/G2 transf	1.27 ± 0.92^a^	1.72 ± 0.96^abc^	0.81 ± 0.96^c^	1.16 ± 0.98^b^	^abc^< 0.05
Biochemical pregnancy	40.71% (160/393)^a^	33.33% (17/51)^b^	27.27 (3/11)	16.66% (1/6)^ab^	ab< 0.05
Clinical pregnancy/cycle	38.67 (152/393)^a^	33.33% (17/51)^b^	27.27% (3/11)	16.66% (1/6)^ab^	^ab^< 0.05
Implantation rate	23.48% (198/843)^abc^	14.28 % (19/133)^ad^	13.04% (3/23)^ce^	6.66% (1/15)^bde^	^acde^< 0.05^b^< 0.01
Singletons	71.05% (108/152)	88.23% (15/17)	100%	100%	
Twins	27.62% (42/152)	11.46% (2/17)	0	0	
Triplets	1.13% (2/162)	0	0	0	
Ectopic pregnancy	0.65% (1/152)	0	0	0	
Abortion	11.84% (18/152)^ab^	47.05% (8/17)^a^	33.33% (1/3)^b^	0	^ab^< 0.05
Life Birth rate/cycle	33.84% (133/393)^abc^	17.64% (9/51)^a^	18.18% (2/11)^c^	16.66% (1/6)^b^	^abc^< 0.05
Stillbirth	1.13% (2/152)	0	0	0	

There was no difference in age, basal FSH and Estradiol, days of stimulation, endometrium thickness and total amount of gonadotropins between groups 1 *versus* group 4. However, significant differences occurred in embryological and clinical outcomes between these two groups.

The intermediate groups (2 and 3) required higher amounts of gonadotrophins when compared to the normal group (1). In fact, both groups (1 and 2) had significant differences in implantation and abortion rates when compared to group 1.

## DISCUSSION

This study evaluates a period of eleven years. A study from Brussels (Kolibianakis *et al*., 2004) related 20 abnormal P4i levels (4.9%) among 410 patients. This led them to postpone the beginning of stimulation in 1-2 days. Notwithstanding, they reported a significantly lower on-going pregnancy per oocyte retrieval. Hamdine *et al*. (2014) carried out a prospective study in combination with a systematic review and meta-analysis, to access cycles. Among 158 patients they reported an incidence of elevated P4i in 13.3% (we had only 3.69% now, and in 2004 it was present in 12% of our patients, 7 out of 60 patients).

During the past few years, discussions have been centered in both tailoring and individualizing protocols and costs, as well as patient convenience during cycles. Listijono *et al*. (2015) observed that higher P4 levels in the beginning of COH resulted in more oocytes. However, no differences in clinical pregnancy rates were found and they even suggested that protocols could just delay the checking of hormonal assays until mid-follicular stimulation.

Our study took a closer look at the progesterone data and found that there is an impact of early *p* levels in antagonist cycles on pregnancies, mainly when this higher P4 levels is also present at the end of the stimulation period. Also, normal progesterone levels resulted in significant better live birth rates than elevated levels, either in the beginning or at the end of the COH.

It is known that the number of oocytes is strongly correlated with the probability of pregnancy and live birth (Sunkara *et al*., 2011). Again, it has been consistently demonstrated that elevated progesterone is an indicator of granulosa cell function, also associated with a higher number of oocytes (Bosch *et al.*, 2010; Venetis *et al*., 2015). Therefore, this condition of elevated progesterone on both the beginning and at the end of COH should not be considered a distressful outcome per se, because those cycles resulted in significantly more recovered oocytes, and it did not depend on the intensity of COH.

However, whatever the mechanisms of progesterone increase in the late follicular phase, its action exerts detrimental effects on the endometrium. High serum progesterone levels on the hCG day of administration are compatible with advanced endometrial histological maturation and differential endometrial gene expression, and both may lead to implantation failure. Another important fact is that progesterone elevation does not seem to be present in frozen-thawed and donor/recipient cycles (Venetis *et al*., 2013). Therefore, a freeze-all strategy can be planned whenever progesterone elevation is detected on COH cycles.

The limitations of our study include the small sample sizes of groups 2, 3 and 4, which could lead to imprecise effect size estimates. But, this is not necessarily a bias. Venetis *et al*. (2016) have just published the probability of using both basal serum progesterone concentration and history of elevated progesterone after COH as predictors of the occurrence of high progesterone levels on the day of hCG, independently of the intensity of ovarian stimulation. In fact, in the last four years there was a trend not to transfer whenever P4 f > 1.5 ng/mL

## CONCLUSION

The impact of serum progesterone in the beginning of stimulation and pregnancy outcomes is a matter of concern. Basal elevated levels could help identify patients that will repeat it on hCG day, being probably a marker to help define a freeze-all strategy to these cycles.
